# Opportunistic Fungal Invasion in COVID-19 Pandemic: A Critical Review in Diagnosis and Management

**DOI:** 10.1055/s-0043-1770921

**Published:** 2023-07-03

**Authors:** Abhishek Sharma, Gulnaz Bano, Abdul Malik, Yuman Rasool, Samrina Manzar, Tarun Singh, Manish Maity

**Affiliations:** 1 Department of Quality Control & Assurance, Hakeem Abdul Hameed Centenary Hospital & Hamdard Institute of Medical Sciences & Research, Jamia Hamdard, New Delhi, India; 2 Department of Pharmacology & Pharmacy Practice, School of Pharmaceutical Education and Research, Jamia Hamdard University, New Delhi, India; 3 Department of Pharmacy Practice, Teerthanker Mahaveer College of Pharmacy, Moradabad, Uttar Pradesh, India; 4 Department of Pharmacy Practice, School of Pharmaceutical Education and Research, Jamia Hamdard University, New Delhi, India; 5 Department of Pharmacy, Maharishi Markandeshwar College of Pharmacy, MM(DU), Mullana, Haryana, India

**Keywords:** SARS-CoV-2, opportunistic fungal infection, mucormycosis, candidiasis, aspergillosis

## Abstract

Severe acute respiratory syndrome corona virus-2 (SARS-CoV-2) is the culprit behind the pandemic across the globe in recent decades. Variants of SARS-CoV especially coronavirus disease 2019 (COVID-19)-related fungus might not be identified or might be misdiagnosed on a worldwide scale. Patients of COVID-19 acquired invasive mycoses, especially if they are very ill or immunosuppressed. Clinical intervention based on various standard guidelines would be necessary to guarantee that *Aspergillus* and *Candida* -infected COVID-19 patients are adequately treated. To facilitate clinical professionals, doctors, paramedics, and laboratory staff in the treatment of various variants of COVID-19 patients with concurrent aspergillosis, candidiasis, mucormycosis, or cryptococcosis, a tabulation format is drafted in this study. We believe it is prudent to take into account the general nature, and variety of the mycosis that is arriving, the strength and limits of the diagnostic tools, clinical conditions, and the need for standardized or customized therapy in various coronavirus-infected patients.

## Introduction

Coronavirus disease 2019 (COVID-19) was initially identified in Wuhan, Hubei Province, China, causing alarm on a global scale as of December 2019. 1 Although the initial wave of the 2019 corona virus and its various variants outbreak have passed, it is present in different parts of the globe. However, opportunistic invasion danger of fungus infection is still quite high. This may be the outcome of a COVID-19-related sickness, which can cause immunological suppression, higher ferritin levels, excessive iron loading, acidosis, endothelium damage, and the requirement for several wide-ranging antibiotics to treat or prevent subsequent infections. In terms of the incidence of invasive fungal infections (IFIs) and the number of persons with diabetes mellitus, India is ranked second among all other countries. In India, particularly in COVID-19, IFIs are probably prevalent. In two to three individuals of COVID-19 individuals, immune suppression causes a decline in T cells like CD4+ and CD8 + . Significant fungal infections were common in critically ill hospitalized patients. 2

### Invasive Mucormycosis

Mucorales fungi-like Rhizopus, Mucor, Rhizomucor, Cunninghamella, and Absidia are responsible for angio-invasive infections known as mucormycosis. 3 Nowadays, the term zygomycosis is used to describe IFI brought on by Zygomycetes. Zygomycetes are molds that reproduce sexually by producing zygospores and have aseptate or pauciseptate, irregularly branched ribbon-like hyphae. They were reclassified as Mucorales and Entomophthorales, respectively, even though these species were previously separated into two orders, Mucorales and Entomophthorales. Entomophthorales molds are rare infections that are generally prevalent in tropical regions and cause chronic sinus and skin conditions that hardly ever affect internal organs. 4 Mucormycosis is prevalent in India with 0.14 / 1000 individuals, which is approximately 80 times greater than the wealthy nations. 5 Depending on where the disease is found, mucormycosis can present differently clinically. 6 Most patients with invasive mucormycosis are immunosuppressed or observed associated with chronic diseases like diabetes mellitus, blood-related malignancies, or have recently undergone a transplant. 4 Patients who have COVID-19 and other variants may be more vulnerable to fungal infections. Mucormycosis is affecting when hyphae enter the circulation and induce thrombosis and necrosis. 6 Mucormycosis infection affects the rhino-orbitocerebral system, skin, and lungs. Although it can also be brought on by another angio-invasive fungus including aspergillosis and *Pseudallescheria boydii* , mucormycosis is the primary cause of the neurological clinical condition known as a rhino-orbitocerebral syndrome. The rhino-orbitocerebral syndrome symptoms show as pain in the face, facial numbness, headache, eye pain, and diplopia with several other eye-related disorders; blackness on the skin and mucosa, along with ulcer and palate drainage. *Mucor* damages necrotic tissue and thrombosis in nearby arteries of the nervous system like the internal carotid artery and cavernous sinus 7 ( Table 1 ).

**Table 1:** Diagnostic and therapeutic pathway for invasive fungal coinfection

COVID-19 patients associated with invasive fungal infections			
1. Severely ill ones (admitted to ICU, required mechanical ventilation, long duration of hospital stay) 2. And/or with an immunocompromised state			
**Invasive fungal infections (IFI)**	**Risk factors**	**Diagnosis**	**Treatment**
Invasive mucormycosis	Trauma, diabetes Mellitus, GC use, allo HSCT, SBT, prolong neutropenia, HM	1. Direct microscopy using fluorescent brightener and histopathology with special stains (PAS, GMS) Typical findings: Nonseptate, ribbon-like hyphae (at least 6–16µm wide) 2. Culture: Routine media 30°C and 37°C Typical finding: cottony white or grayish black colony. 3. Molecular Identification: PCR-based assays, HRM target gene:18S, ITS,28S or rDNA	1. Surgical treatment: If possible 2. Primary Prophylaxis: Posaconazole 3. First-line treatment: amphotericin B lipid complex, liposomal amphotericin B, posaconazole oral suspension
Invasive candidiasis	Parenteral nutrition, Broad spectrum antibacterial drug use, Invasive examinations	1. Direct Microscopy using Calcofluor or Blankophor Typical finding: Pseudohyphae 2. Culture: Blood or other sterile samples Typical findings: cream like 3. Serology: Mannan and anti-mannan IgG tests, CAGTA, BDG 4. Molecular identification: PCR-based assays, target gene: r-DNA, ITS 5. New methods: T2 magnetic resonance and MALDI-TOF technology	1 Echinocandin (caspofungin, Micafungin, anidulafungin) 2 Triazoles (fluconazole, voriconazole, itraconazole) 3 Amphotericin B and its liposomes
Invasive aspergillosis	GC use, COPD, prolonged neutropenia, allo-HSCT, SBT, inherited immunodeficiencies, CF, HM	1. Direct microscopy using Calcofluor or Blankophor and histopathology with special stains (PAS, GMS) Typical findings: acute angle branching septate hyphae 2. Culture: 37° C for 2–5 days, morphological features 3. Molecular Identifications: PCR-based assays, target gene: Ben-A, CAL, and ITS 4. GM test: Serum and BALF	1. Triazoles (voriconazole, posaconazole, isocoanazole, itraconazole) 2. Amphotericin B and its liposomes 3. Echinocandin (micafungin, caspofungin)
Invasive Cryptococcosis	HIV infection (CD4 < 200 cells/µL), allo-HSCT, SOT	1. Direct microscopy: CSF mixed with India ink, narrow budding encapsulated yeasts 2. Culture: 30°C for 7 days, in aerobic conditions, mucoid creamy colonies 3. Serology: CrAg, LAT, EIA, LFA 4. Molecular identification: Pan fungal PCR, DNA sequencing, multiplex PCR, isothermal amplification, probe-based micro-assays, target gene: IGS1, CAP5, ITS	1. Induction Phase: Amphotericin B deoxycholate and flucytosine followed by fluconazole; alternative for fluconazole+ flucytosine or amphotericin B deoxycholate + fluconazole. 2. Consolidation phase: fluconazole 3. Maintenance phase: fluconazole

Abbreviations: BALF, bronchoalveolar lavage fluid; CF, cystic fibrosis; COPD, chronic obstructive pulmonary disease; COVID-19, coronavirus disease 2019; CSF, cerebrospinal fluid; EIA, enzyme-linked immunoassay; GM, galactomannan; GMS, Grocott-Gomori's methenamine-silver; ICU, intensive care unit; HIV, human immunodeficiency virus; HM, haematological malignancy; allo-HSCT, allogeneic hematopoietic stem cell transplantation; LAT, latex agglutination test; PAS, periodic acid-Schiff; PCR, polymerase chain reaction; SBT, solid body transplantation.

### Diagnosis

It is recommended to employ computed tomography (CT) or magnetic resonance imaging (MRI) to see the brain, intracranial arteries, and paranasal sinuses. The first imaging method often used a CT scan that can detect bone dehiscence or disintegration. MRI can detect vascular invasion, an intracranial tumor, and the optic nerve. 8 Potassium hydroxide (KOH) and Calcofluor, two fungus-specific stains/culture, histopathology, and molecular diagnostic with polymerase chain reaction (PCR), are used in the research. 9

### Management

Therapeutic management of fungal infections acquired by COVID-19 and its other variants with reference to standards are required to deliver antifungal medications and surgical management. Moreover, this opportunistic infection is also associated to acquire promptly metabolic abnormalities management that increases the mortality risk of mucormycosis by 50%.

### Medical Management

Diagnostically, it is necessary to record MRI or CT scan to observe the brain, intracranial arteries, and paranasal sinuses. The first imaging method often used, a CT scan, can detect bone dehiscence or disintegration. MRI can detect vascular invasion, an intracranial tumor, and the optic nerve. 8 KOH and Calcofluor, fungus-specific antibiogram and molecular diagnostic test should be ordered before confirming the diagnosis.

Medical management of this opportunistic infection required liposomal amphotericin B/amphotericin deoxycholate and standard amphotericin B/amphotericin deoxycholate that are effective and have more unfavorable side effects. 10 Amphotericin is commonly infused over a period of 1 to 4 hours at a rate of 0.3 to 1.5 mg/kg/day. Before starting intravenous therapy, a 1-mg test dosage should be given. People with adequate renal function can receive amphotericin B over the course of 1 to 2 hours. 11 Two other drugs for the treatment of mucormycosis include posaconazole (300 mg twice a day for 3 days, then 300 mg daily, orally) and isavuconazole(200 mg twice daily on the first day followed by 200 mg daily). 12 Isavuconazole demonstrated efficacy comparable to amphotericin in an open-label research. 13 For the oral formulation, a long shelf life of up to 180 days is available. 14 Antifungal drugs including caspofungin, voriconazole, and fluconazole cannot treat mucormycosis ( Table 1 ).

### Surgical Management

Osteomyelitis abscess, debridement, and various sorts of necrotic tissue removal can all be handled surgically. The surgical team should include an ENT specialist, an eye specialist, a dental surgeon, and a neurosurgeon, depending on the area that is impacted. Mucormycosis is frequently treated with orbital debulking and functional endoscopic sinus surgery. Since medications cannot reach necrotic tissue, early surgery is better than late surgery. Patients with mucor require substantial surgical help in order to have a decent prognosis. Depending on how the patient reacts to the treatment, a check endoscopy or surgical inquiry may need to be repeated in some circumstances. Uncertain and dependent on the specifics of each case, the length of therapy will be determined. Amphotericin therapy is recommended for 4 to 6 weeks. For a few more weeks, posaconazole or isavuconazole might be used as maintenance therapy 15 ( Table 1 ).

### Invasive Candidiasis

The most common yeast species are identified on mucosal surfaces, such as respiratory, digestive, and urinary systems. The most commonly isolated pathogens included *Candida albicans* , *Candida glabrata* , *Candida krusei, Candida parapsilosis* , and *Candida tropicalis* . The most common cause of invasive candidiasis appears to be *Candida albicans* . 16 COVID-19 patients, who are severely ill and treated with antiretroviral medicines, parental meals, and intrusive testing, as well as those with persistent neutropenia and other physical limitations, may be at higher risk of *Candida* -type infection. The primary cause of fatal illnesses and one of the components of the human mycobiome are thought to be *Candida* species. The deleterious effects of severe COVID-19 are increasingly being recognized as invasive yeast infections. 17 The risk factors for the illness have expanded, and now include persons who have undergone solid organ and hematopoietic stem cell transplantation (HSCT), used immunosuppressive medications, had HIV infection, were prematurely born, were older, underwent surgery, or had cancer. 18 The most frequent clinical symptom of invasive candidiasis is bloodstream infection with a *Candida species* (candidemia), which is an important source of rising sentinel events and death for hospital-admitted patients. 16 Septic shock is a clinical sign of candidemia in affected persons. According to one study, people with *Candida* spp. ocular shock is more likely to experience renal and hepatic failure and had lower levels of lactic dehydrogenase than those with bacterial shock. Ones connected to internal and external candidiasis include abscesses, peritonitis, pancreatitis, and cholangitis 19 ( Table 1 ).

### Diagnosis

Without initially understanding the extent of the disease, the results of an invasive candidiasis diagnostic test cannot be properly interpreted. Invasive candidiasis can manifest as either one of two forms, candidemia, or severe candidiasis. 20 The detection limit for live *Candida* in blood cultures is equivalent to or superior to that of PCR. Cerebrospinal fluid (CSF) samples are reliable indicators of candida meningitis in patients. Nonculture diagnostic techniques for invasive candidiasis include mannan, anti-mannan antibody, and *C. albicans* germ tube antibody. Initially, diagnosis with non-culture for invasive candidiasis was serum tests for *Candida* antigens and anti- *Candida* antibodies. Most *Candida* antigens have limited diagnostic use due to low serum concentrations and fast circulatory clearance. Numerous cell wall components such as mannan and 1,3-D-glucan (BDG) are the most efficient targets. 21 The T2 *Candida* panel is an additional diagnostic marker, and BDG is an essential part of the *Candida* cell wall and the fungus infection. T2 *Candida* nanodiagnostic panel has received Food and Drug Administration approval for the detection of candidemia. To find *Candida* in whole blood, T2 *Candida* employs an automated method that makes use of K2 EDTA Vacutainer collection tubes and a customized equipment platform (T2Dx) 22 ( Table 1 ).

### Management

A higher mortality rate has been observed in COVID-19 patients with IFIs. Timely identification and treatment are essential for a positive clinical result as compared to individuals who did not get antifungal medication. Invasive candidiasis therapy in COVID-19 patients is the same as for people without the virus. 23 Echinocandins, azoles, and polyenes are now the three steps of therapy for candidiasis. Additionally, the pyrimidine analogue flucytosine has a unique place in the management of *Candida endocarditis* and intermediate candidiasis. 24 25 Pharmacological regimen for invasive *Candida* infections involves echinocandins firstly, then liposomal amphotericin B, fluconazole, posaconazole, voriconazole, and isavuconazole. 17 18 19 20 21 22 23 26 27 28

### Invasive Aspergillosis

COVID-19 patients who have high-risk factors for life-threatening infections are particularly susceptible to *Aspergillus* consequences. 29 30 Allogeneic hematopoietic stem cell transplant (allo-HSCT), solid body transplantation (SBT), inherited disability, use of gas chromatography (GC), chronic neutropenia, chronic obstructive pulmonary disease (COPD), SBT, and diseases (acute respiratory distress syndrome [ARDS]) resulting from viral infections tend to secondary problems such as noninvasive aspergillosis even with a well-defined immune system. 31 32 Hypoxic risk of internal defenses and autoimmune illnesses brought on by ARDS are two possible causes for this 33 ( Table 1 ).

### Diagnosis

Distinguishing *Aspergillus* species from other filamentous fungi ( *Fusarium* species and *Scedosporium* species) can be challenging; histopathologic tests based on spotting specific fungal areas in fluid or tissue, where there is suspicion of fungal infection, may reveal a critical septate hypertension feature of *Aspergillus* species. Additionally, Grocott-Gomori's methenamine-silver and periodic acid-Schiff of organized tissue will help. 34 Therefore, we need to have descriptive evidence of cultural or nontraditional techniques, including (i) direct testing of optical light, Calcofluor or Blankophor light, which may increase sensitivity and clarity of detection similar to *Aspergillus* ; (ii) culture in fungal sources at 37 °C 2 to 5 days, if positive, morphological features of *Aspergillus* can be detected under a microscope or DNA sequences can be used in laboratory indicators for accurate diagnosis, though low culture is seen and side effects do not exclude immunoassay (IA) diagnosis; (iii) molecular testing based on ribosomal DNA sequence (rDNA) can also be used to detect *Aspergillus* tissue or bronchoalveolar lavage fluid (BALF), especially PCR trials may be used to detect *Aspergillus* spp. and conversion of CYP51A resistance to A. Fumigatus, although these methods are not limited by laboratory conditions or reagents; 35 (iv) serum and BALF GM tests are also recommended as early and accurate diagnosis using negative diagnostic procedures, especially in non-neutropenia patients, who have the benefit of minor injury and long-term functionality. Sometimes these blood sample tests are less sensitive than respiratory sampling cultures 36 ( Table 1 ).

### Management

Typically, triazoles (itraconazole, voriconazole, posaconazole, and isavuconazole), amphotericin B, and its liposomes are used for the treatment and prevention of IA (micafungin or caspofungin). However, drug monitoring is advised. Besides, the interaction between azoles and other medications ought to be taken into account, carefully. The majority of individuals may select triazole medications to treat IA ( Table 1 ).

### Invasive *Cryptococcosis*

*Cryptococcus neoformans* or *Cryptococcus gattii* are the pathogens that cause crypto coccidiosis, an invasive tuberculosis that is still spreading around the world. 37 Individuals with cryptococcosis are frequently immobile, and many of them had previously tested positive for HIV. However, there have also been accounts of patients who are believed to be frail. 38 Meningoencephalitis has been linked to infections with *Cryptococcus neoformans* in individuals who are not responding. 39 However, it occurs less frequently in impotent individuals, such as HIV patients linked with CD4 and COVID-19. The most typical symptom of cryptococcosis is meningoencephalitis, which is caused by immunological diseases such as T-lymphocyte 200 cells/ *μ* L, allo-HSCT, SBT, or others 17 39 40 ( Table 1 ).

### Diagnosis

*Cryptococcus* species findings that include *Cryptococcus neoformans* and *C types* led to the finding of cryptococcosis. Gattii, an amalgamation of clinical as well as laboratory validation, is often used to make the diagnosis of cryptococcosis. Histopathology, serology, cell detection, and exact microscopy are techniques used to confirm culture infection. A little amount of frequently found blended yeast can be added to a sample of CSF to develop a specific structure for *Cryptococcus* spp. to diagnose cryptococcosis. Cultural samples need to be incubated in Sabouraud dextrose agar at 30° C for 7 days while being examined every day under aerobic circumstances. Additionally, cultures may take longer to grow in individuals on systemic antifungal therapy. The green coin of the coin is thought to be *Cryptococcus* serum, CSF, BAL, or diseased tissues can be used to collect and quantify the capsular polysaccharides of *Cryptococcus* . There are now three different cryptococcal antigens tests available: lateral flow immunoassay (LFI), enzyme-linked immunoassay (EIA), and latex agglutination test (LAT). The BAL, pleural fluid, and sputum samples used in respiratory samples, such as testing, are not suitable for these rapid, sensitive, and accurate procedures. 41 In rare instances, cell identification is necessary to validate cryptococcosis diagnosis when other diagnostic techniques fail to work. Pan-fungal PCR, DNA sequencing detection, multiplex PCR, isothermal pathway magnification, and investigative microarrays are some of these molecular processes. Lumbar and CSF testing, further including antigen, are advised for patients as soon as cryptococcosis is diagnosed ( Table 1 ).

### Management

Drugs in the ensuing forms are suggested as desirable ones in the intake phase of flucytosine or amphotericin B deoxycholate followed by fluconazole; these are additional fluconazole treatment alternatives ( Table 1 ).

## Discussion

Depending on the fungus that is infected, IFI puts patients who have co-morbidities at a significant chance of dying. IA is most frequently seen in neutropenic patients, receiving chemotherapy, has severe hematological conditions, are receiving long-term corticosteroid therapy or biotherapy, have solid tumors or HSCT allografts, or have chronic respiratory conditions. Pneumocystosis is a contagious illness that can affect people with lymphopenia, HIV, hematological problems, severe or persistent respiratory infections, and lymphopenic individuals. Most cases of infectious mucormycosis are associated with individuals who have diabetes, severe hematological disorders, solid organ transplants, chronic respiratory illnesses, persistent burn injuries, or post-traumatic stress disorder. The number of COVID-19 patients being managed is growing swiftly, and real-time testing is being used to quickly provide tailored medication. Aspergillosis, pneumocystosis, and mucormycosis have significantly different first-line therapies, and these therapies will be avoided wherever feasible. Preventive strategies like antifungal chemoprophylaxis and natural remedies might be taken into consideration to lower morbidity and death based on the epidemiological data that is currently available ( Table 1 ).

## Conclusion

**Fig. 1:**
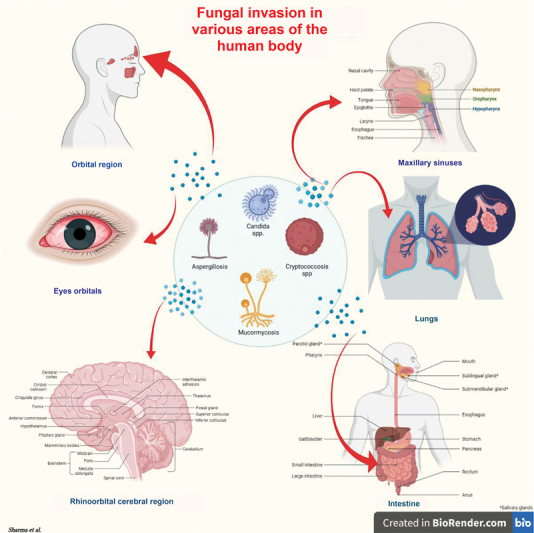
Fungal infections affecting major organs of the body.

We predict that the widespread COVID-19 cofungal infection might be failed to notice or would have made an incorrect diagnosis. Additionally, as a potentially fatal infectious condition, patients with COVID-19 have excessive suppression of inflammatory cytokines and impaired immune response by having reduced CD4 T and CD8 T cell counts, which allowed for the discovery of a fungal coinfection. Additionally, COVID-19 patients often have other autoimmune conditions such as chronic neutropenia, HSCT, GC, SBT, hereditary conditions, or herbal medicines. The tumor may also encourage a joint fungal infection. The revised diagnostic information (histopathology, extremely tiny tests, culture, (1,3) -bD-glucan, galactomannan, PCR tests, matrix-assisted laser desorption/ionization-time of flight [MALDI-TOF] technology, etc.) and noninvasive mycosis therapy suggestions are outlined here. We advise that it is wise enough to consider risk factors and different forms of incurable mycosis, the benefits and drawbacks of diagnostic procedures, clinical settings, and the requirement for standard or unique care for COVID-19 patients. To help doctors and lab experts manage aspergillosis, candidiasis, mucormycosis, or cryptococcosis as associated illnesses in patients with COVID-19, we have supplied a tabulation table ( Table 1 ). A quick explanation of the types of mucor infections that affect different organ systems in the human body has been provided in the form of a diagram ( Fig. 1 ).

## References

[1] China Medical Treatment Expert Group for Covid-19 Guan W J Ni Z Y Hu Y Clinical characteristics of coronavirus disease 2019 in China N Engl J Med 2020 382 18 1708 1720 32109013 10.1056/NEJMoa2002032 PMC7092819

[2] Song G Liang G Liu W Fungal co-infections associated with global COVID-19 pandemic: a clinical and diagnostic perspective from China Mycopathologia 2020 185 04 599 606 32737747 10.1007/s11046-020-00462-9 PMC7394275

[3] Revannavar S M Samaga L COVID-19 triggering mucormycosis in a susceptible patient: a new phenomenon in the developing world? BMJ Case Rep 2021 14 04 e241663 10.1136/bcr-2021-241663 PMC8088249 33906877

[4] Farmakiotis D Kontoyiannis D P Mucormycoses Infect Dis Clin North Am 2016 30 01 143 163 26897065 10.1016/j.idc.2015.10.011

[5] Skiada A Pavleas I Drogari-Apiranthitou M Epidemiology and diagnosis of mucormycosis: an update J Fungi (Basel) 2020 6 04 265 33147877 10.3390/jof6040265 PMC7711598

[6] Monte Junior E SD Santos M ELD Ribeiro I B Rare and fatal gastrointestinal mucormycosis (Zygomycosis) in a COVID-19 patient: a case report Clin Endosc 2020 53 06 746 749 33207116 10.5946/ce.2020.180 PMC7719411

[7] Kulkarni R Misra U K Meshram C Epidemic of mucormycosis in COVID-19 pandemic: a position paper Ann Indian Acad Neurol 2022 25 01 7 10 35342268 10.4103/aian.AIAN_421_21 PMC8954338

[8] Safder S Carpenter J S Roberts T D Bailey N The “Black Turbinate” sign: an early MR imaging finding of nasal mucormycosis AJNR Am J Neuroradiol 2010 31 04 771 774 19942703 10.3174/ajnr.A1808 PMC7964235

[9] Millon L Scherer E Rocchi S Bellanger A P Molecular strategies to diagnose mucormycosis J Fungi (Basel) 2019 5 01 24 30897709 10.3390/jof5010024 PMC6463105

[10] Cagnoni P J Liposomal amphotericin B versus conventional amphotericin B in the empirical treatment of persistently febrile neutropenic patients J Antimicrob Chemother 2002 49 01 81 86 11801587 10.1093/jac/49.suppl_1.81

[11] Kintzel P E Smith G H Practical guidelines for preparing and administering amphotericin B Am J Hosp Pharm 1992 49 05 1156 1164 1595747

[12] Mucormycosis ECMM MSG Global Guideline Writing Group Cornely O A Alastruey-Izquierdo A Arenz D Global guideline for the diagnosis and management of mucormycosis: an initiative of the European Confederation of Medical Mycology in cooperation with the Mycoses Study Group Education and Research Consortium Lancet Infect Dis 2019 19 12 e405 e421 31699664 10.1016/S1473-3099(19)30312-3 PMC8559573

[13] VITAL and FungiScope Mucormycosis Investigators Marty F M Ostrosky-Zeichner L Cornely O A Isavuconazole treatment for mucormycosis: a single-arm open-label trial and case-control analysis Lancet Infect Dis 2016 16 07 828 837 26969258 10.1016/S1473-3099(16)00071-2

[14] Unnikrishnan R Anjana R M Mohan V Diabetes mellitus and its complications in India Nat Rev Endocrinol 2016 12 06 357 370 27080137 10.1038/nrendo.2016.53

[15] Kontoyiannis D P Lewis R E How I treat mucormycosis Blood 2011 118 05 1216 1224 21622653 10.1182/blood-2011-03-316430 PMC3292433

[16] Pappas P G Lionakis M S Cavling Arendrup Maiken Ostrosky-Zeichner L Jan Kullberg B Invasive candidiasis; a review article Jundishapur J Microbiol 2018 11 04 18026 10.1038/nrdp.2018.26 29749387

[17] Arastehfar A Carvalho A Nguyen M H COVID-19-Associated candidiasis (CAC):an underestimated complication in the absence of immunological predisposition? J Fungi (Basel) 2020 6 04 211 33050019 10.3390/jof6040211 PMC7712987

[18] Morse S S Factors and determinants of disease emergence Rev Sci Tech 2004 23 02 443 451 15702712 10.20506/rst.23.2.1494

[19] Antinori S Milazzo L Sollima S Galli M Corbellino M Candidemia and invasive candidiasis in adults: a narrative review Eur J Intern Med 2016 34 21 28 27394927 10.1016/j.ejim.2016.06.029

[20] Clancy C J Nguyen M H Finding the "missing 50%" of invasive candidiasis: how nonculture diagnostics will improve understanding of disease spectrum and transform patient care Clin Infect Dis 2013 56 09 1284 1292 23315320 10.1093/cid/cit006

[21] Ellepola A N Morrison C J Laboratory diagnosis of invasive candidiasis J Microbiol 2005 43(Spec No):65 84 15765060

[22] Pfaller M A Moet G J Messer S A Jones R N Castanheira M Candida bloodstream infections: comparison of species distributions and antifungal resistance patterns in community-onset and nosocomial isolates in the SENTRY Antimicrobial Surveillance Program, 2008-2009 Antimicrob Agents Chemother 2011 55 02 561 566 21115790 10.1128/AAC.01079-10 PMC3028787

[23] Koehler P Arendrup M C Arikan-Akdagli S European Confederation of Medical Mycology (ECMM). ECMM CandiReg-A ready-to-use platform for outbreaks and epidemiological studies Mycoses 2019 62 10 920 927 31271702 10.1111/myc.12963 PMC7614793

[24] Ben-Ami R Treatment of invasive candidiasis: a narrative review J Fungi (Basel) 2018 4 03 97 30115843 10.3390/jof4030097 PMC6162658

[25] Nguyen M H Yu V L Meningitis caused by Candida species: an emerging problem in neurosurgical patients Clin Infect Dis 1995 21 02 323 327 8562739 10.1093/clinids/21.2.323

[26] Clancy C J Nguyen M H Diagnosing invasive candidiasis J Clin Microbiol 2018 56 05 e01909 e01917 29444828 10.1128/JCM.01909-17 PMC5925725

[27] Lyons J L Zhang S X Current laboratory approaches to diagnosis of CNS fungal infections Future Microbiol 2016 11 02 175 177 26849164 10.2217/fmb.15.138

[28] White P L Dhillon R Cordey A A national strategy to diagnose COVID-19 associated invasive fungal disease in the ICU Clin Infect Dis 2021 73 07 e1634 e1644 32860682 10.1093/cid/ciaa1298 PMC7499527

[29] Miceli M H Churay T Braun T Kauffman C A Couriel D R Risk factors and outcomes of invasive fungal infections in allogeneic hematopoietic cell transplant recipients Mycopathologia 2017 182(5-6):495 504 28124219 10.1007/s11046-017-0115-y

[30] Fishman J A Grossi P A Novel Coronavirus-19 (COVID-19) in the immunocompromised transplant recipient: #Flatteningthecurve Am J Transplant 2020 20 07 1765 1767 32233057 10.1111/ajt.15890 PMC7228206

[31] Poli P Timpano S Goffredo M Padoan R Badolato R Asymptomatic case of Covid-19 in an infant with cystic fibrosis J Cyst Fibros 2020 19 03 e18 32303430 10.1016/j.jcf.2020.03.017 PMC7152906

[32] Koehler P Cornely O A Böttiger B W COVID-19 associated pulmonary aspergillosis Mycoses 2020 63 06 528 534 32339350 10.1111/myc.13096 PMC7267243

[33] Jamieson A M Yu S Annicelli C H Medzhitov R Influenza virus-induced glucocorticoids compromise innate host defense against a secondary bacterial infection Cell Host Microbe 2010 7 02 103 114 20159617 10.1016/j.chom.2010.01.010 PMC2836270

[34] AspICU Study Investigators Blot S I Taccone F S Van den Abeele A M A clinical algorithm to diagnose invasive pulmonary aspergillosis in critically ill patients Am J Respir Crit Care Med 2012 186 01 56 64 22517788 10.1164/rccm.201111-1978OC

[35] Patterson T F Thompson G R III Denning D W Practice guidelines for the diagnosis and management of aspergillosis: 2016 update by the Infectious Diseases Society of America Clin Infect Dis 2016 63 04 e1 e60 27365388 10.1093/cid/ciw326 PMC4967602

[36] Hage C A Carmona E M Epelbaum O Microbiological laboratory testing in the diagnosis of fungal infections in pulmonary and critical care practice. An official American Thoracic Society clinical practice guideline Am J Respir Crit Care Med 2019 200 05 535 550 31469325 10.1164/rccm.201906-1185ST PMC6727169

[37] Perfect.Microbiology and epidemiology of Cryptococcus neoformans infection[May;2020]. GMCoxJ... R.2020

[38] Passerini M Terzi R Piscaglia M Passerini S Piconi S Disseminated cryptococcosis in a patient with metastatic prostate cancer who died in the coronavirus disease 2019 (COVID-19) outbreak Cureus 2020 12 05 e8254 32596073 10.7759/cureus.8254 PMC7309194

[39] Khatib M Y Ahmed A A Shaat S B Mohamed A S Nashwan A J Cryptococcemia in a patient with COVID-19: a case report Clin Case Rep 2020 9 02 853 855 33598258 10.1002/ccr3.3668 PMC7869327

[40] Setianingrum F Rautemaa-Richardson R Denning D W Pulmonary cryptococcosis: a review of pathobiology and clinical aspects Med Mycol 2019 57 02 133 150 30329097 10.1093/mmy/myy086

[41] Ibáñez-Martínez E Ruiz-Gaitán A Pemán-García J Update on the diagnosis of invasive fungal infection Rev Esp Quimioter 2017 30 01 16 21 28882009

